# Increased symptoms of post-traumatic stress in school students soon after the start of the COVID-19 outbreak in China

**DOI:** 10.1186/s12888-021-03339-3

**Published:** 2021-07-03

**Authors:** Hanmei Xu, Hang Zhang, Lijuan Huang, Xiaolan Wang, Xiaowei Tang, Yanping Wang, Qingqing Xiao, Ping Xiong, Rongqiu Jiang, Jie Zhan, Fang Deng, Mingya Yu, Dong Liu, Xuejun Liu, Chunli Zhang, Wenjun Wang, Lu Li, Hongmei Cao, Wenchao Zhang, Hongping Zhou, Wo Wang, Li Yin

**Affiliations:** 1 Mental Health Center, West China Hospital of Sichuan University, No. 28 South Dianxin Street, Chengdu, 610041 Sichuan China; 2 Chengdu Engineering Technical Vocational School, Chengdu, 610300 Sichuan China; 3 Chengdu Vocational & Technical College of Industry, Chengdu, Sichuan 610218 China; 4 Xuchuan Middle School, Zigong, 643000 Sichuan China; 5 The Fourth People’s Hospital of Chengdu, Chengdu, 610036 Sichuan China; 6 Jiangsu Academy of Educational Sciences, Nanjing, 210013 Jiangsu China; 7 Wenquan Second Central Primary School, Donghai County, Lianyungang, 222315 Jiangsu China; 8 Jiangsu Shuangdian Primary School, Rudong County, Nantong, 226404 Jiangsu China; 9 The Sixth Middle School of Jianshui County, Honghe Prefecture, Jianshui County, 654300 Yunnan China; 10 Egongyan Primary School, Jiulongpo District, Chongqing, 404000 China; 11 The 12th Elementary School of Nanyang City, Nanyang, 473002 Henan China; 12 Hou Central School, Xuzhou, 221000 Jiangsu China; 13 The primary School Attached to SouthWest University, Chongqing, 400700 China; 14 Chengdu Shuangliu Yongan Middle School, Chengdu, 610219 Sichuan China; 15 University-Town Hospital of Chongqing Medical University, Chongqing, 401331 China; 16 Institute for System Genetics, Frontiers Science Center for Disease-related Molecular Network, Chengdu, 610041 Sichuan China

**Keywords:** Post-traumatic stress, COVID-19, School students, CRIES-13, Stress, China

## Abstract

**Background:**

The outbreak of Coronavirus Disease 2019(COVID-19) caused psychological stress in Chinese adults population. But we are unaware of whether the pandemic causes psychological stress on children.

**Methods:**

We used the Children’s Impact of Event Scale questionnaire (CRIES-13) to investigate the degree of Post-traumatic Stress (PTSD) symptoms caused by the pandemic in students selected from schools in Sichuan, Jiangsu, Henan, Yunnan, and Chongqing provinces of China.

**Results:**

A total of 7769 students(3692 male and 4077 female), aged 8–18 years, were enrolled in the study, comprising 1214 in primary schools, 2799 in junior high schools and 3756 in senior high schools. A total of 1639 students (21.1%) had severe psychological stress reactions. A large proportion of senior high school students (23.3%) experienced severe psychological stress, and they had the highest median total CRIES-13 score. Female students were more likely to experience severe psychological stress and had higher median CRIES-13 total scores than males.

**Conclusion:**

COVID-19 has placed psychological stresses on primary and secondary school students in China. These stresses are more likely to reach severe levels among female students and senior high school students.

## Background

Studies have shown an increased incidence of post-traumatic stress in survivors of large-scale disasters compared with the general population [1–4]. Disasters can be defined as destructive occurrences that disrupt and overwhelm entire communities and affect millions worldwide in a given year [5]. Children who have experienced disasters are more vulnerable than adults to mental and psychological disorders, including post-traumatic stress disorder (PTSD) [6–8]. Disasters can severely affect their emotional status, resulting in stress reactions that are different from those experienced by adults. Unlike adults who are able to regulate their emotions, children are more likely to limit or suppress their emotions [9, 10]. In addition, children may develop serious psychological and mental illnesses that occur sooner and last longer than those in adults [11–13]. And there is ample evidence of gender differences in post-traumatic symptomatology and women are found more likely to develop symptoms than men [3].

In the twenty-first century, a number of infectious diseases have challenged global public health [14].

During the epidemic of severe acute respiratory syndrome in February 2003, many adult patients developed post-traumatic stress symptoms, PTSD, anxiety, depression, and other mental illnesses [15–18]. The current Coronavirus Disease 2019(COVID-19) pandemic also has constituted a global public health disaster [19]. After COVID-19 outbreak in China, our governments issued the first-level public health alert and recommended that all citizens staying at home [20]. The Ministry of Chinese Education estimated that more than 220 million children and adolescents were confined to their homes. In such crisis time, it is necessary to explore whether the COVID-19 pandemic would cause psychological stress on children and adolescents.

Therefore, we investigated the prevalence of post-traumatic stress symptoms in primary and secondary school students from several provinces and regions in China at one month after the start of the COVID-19 outbreak in order to elucidate the effects of the pandemic on the psychological stress in children and adolescents.

## Methods

### Subjects

We recruited 7769 students, from those in first grade of primary school (8 years) to those in the third grade of senior high school (18 years), in Sichuan(6727 students, 2 elementary school, 3 middle school), Jiangsu(767 students, 3 elementary school,1 middle school), Shandong(159 students, 1 elementary school,1 middle school), Henan(10 students, 1 elementary school), Yunnan(43 students, 1 middle school), and Chongqing(63 students, 2 elementary school). Participants were stratified into primary school students (grades1–6), junior high school students (grades7–9), and senior high school students (grades10–12). We excluded students with a history of substance abuse and those suffering from mental illnesses (totally 4.5%), as well as those who could not understand the questionnaire.

The minimal sample size required for this study was calculated based on the typical sample size for questionnaire-based surveys of the occurrence of post-traumatic stress after disasters. Based on a PTSD prevalence of 32.2% in China after the outbreak of COVID-19 [19], we calculated a minimal sample of 2097 for a power of 0.8, type I error of 0.05 and allowable error of 0.02. We increased this by 10% to 2330 to compensate for missing or uncooperative participants. Ultimately, our sample was much larger (7769).

### Measurement

Between 1 February 20 and 1 March 12,020, approximately one month after the outbreak of COVID-19 in China, we collected demographic data including age, sex, grade, family structure, occupation of parents and family members, etc. Psychopathological data was collected using the Children’s Revised Impact of Event Scale (CRIES) [21]. After obtaining the informed consent of the participants and their parents, the questionnaire were distributed by parents. Questionnaires couldn’t be submitted until they completed all questions, so there is no missing value in our sample.

The CRIES-13 measures symptoms of intrusion (4 items), avoidance (4 items), and arousal (5 new items). Answer item is set as “not at all”, “rarely”, “sometimes”, and “often” [21, 22] . The CRIES-13 total score is used to judge the severity of the psychological impact caused by a traumatic event. A total score ≥ 30 is considered to indicate severe psychological stress [22–24].

### Statistical analysis

All statistical analyses were performed using SPSS 25.0(IBM, Armonk, NY, USA), and the significance level was set as α = 0.05.We analyzed participant data and compared CRIES-13 scores across groups using the Chi-squared, Mann-Whitney U, and Kruskal-Wallis H tests. Post-hoc comparisons were conducted after adjusting the level of significance using Bonferroni correction.

We performed stepwise binary logistic regression using the forward likelihood ratio (LR) method in order to identify factors influencing perceived stress. We considered the influence of sex, age, grade, family structure, occupation of parents, past history of psychological illness (history of psychological consultations or use of psychotropic drug therapy), recent diagnosis of COVID-19, and exposure to coronavirus infection within the previous 30 days. Questions about infection exposure addressed the number of visits to Hubei province and surrounding areas, contact with patients diagnosed with COVID-19, incidence/occurrence of cold, fever, cough, nasal congestion, runny nose, sore throat, and diarrhea, and participation in large gatherings, such as dinner parties. Exposure was also assessed based on contact with family members who were doctors and frontline workers, as well as relatives within three generations who had been diagnosed with COVID-19 or were suspected of COVID-19. We also included data on whether participants had received therapy against COVID-19, or had fever and other mild symptoms.

In order to reduce information bias, we used blind method (blinded to data analyst) to collect data, and two psychiatrists carried out strict quality control of the questionnaire. Because we strictly enforce the inclusion criteria and exclusion criteria, there are such restrictions on participants to avoid confounding bias due to other diseases. At the same time, our study did logical regression analysis to minimize the impact of confounding factors.

## Results

### Demographic and clinical characteristics of subjects

Our survey included a total of 7769 students (4077 female) from 5 different provinces in China (Table 1). All participants were between 8 and 18 years old (median 15 years), and were stratified into three groups based on their grade: primary school students (15.62%, median age 11 years), junior high school students (36.03%, median age 13 years), and senior high school students (48.35%, median age 16 years). In total, 24 participants (13 males and 11 females) were diagnosed with COVID-19, and 27 (16 males and 11 females) were suspected of being infected with the disease. There were significant differences in gender distribution among different grades(*x*^2^ = 32.234, *p* < 0.001).

**Table 1:** Demographic characteristics of school students, stratified by sex and grade

	Total (***n*** = 7769)	Sex				Grade				
Characteristic		Male (***n*** = 3692)	Female (***n*** = 4077)	Z/× 2	p	Primary (***n*** = 906)	Junior High (***n*** = 2799)	Senior High (***n*** = 3756)	H/x2	p
**Median age** (years)	15	15	15	−1.910	0.056*	11.00	13.00	16.00	6159.783	0.000*
**Sex** (Male/Female)	–	–	–	–	–	611/603	1420/1379	1661/2095	32.234	0.000*
**Grade** (Primary school/Junior high school/Senior high school)	1214/2799/3756	160/451/1420/1661	148/455/1379/2095	32.324	0.000*	–	–	–	–	–
**Family structure**				50.179	0.000*				384.706	0.000*
Single parent	794	346	448			72	252	470		
Two parents	2608	1339	1269			160	1064	1384		
Three-generation	2762	1350	1412			601	952	1209		
Other	1605	657	948			381	531	693		
**Occupation of parents**				34.448	0.000*				781.740	0.000*
Medical staff	160	86	74			7	108	45		
Police	58	31	27			0	40	18		
Civil servant	287	151	136			7	164	116		
Teacher	195	102	93			12	114	69		
Freelancer	1838	906	932			270	637	931		
Farmer	1073	477	596			349	185	539		
Researcher	12	8	4			0	8	4		
Worker	1575	677	898			299	360	916		
Self-employed	1586	797	789			166	761	659		
Others	985	457	528			104	422	459		
**Family members**										
Infected (Yes/No)	165/1604	70/3622	95/3982	1.757	0.185	9/1205	56/2743	100/3656	16.607	0.000*
Doctor (Yes/No)	272/7497	145/3547	127/3950	3.785	0.052	14/1200	166/2633	92/3664	81.008	0.000*
Frontline worker (Yes/No)	111/7658	51/3641	60/4017	0.112	0.738	18/1196	66/2733	27/3729	30.627	0.000*
**Respondent diagnosed with COVID-19**	24/7745	13/3679	11/4066	0.426	0.514	6/1208	4/2795	14/3742	4.355	0.113
**Respondent had contact with suspected COVID-19 patient** (Yes/No)	27/7742	16/3676	11/4066	1.497	0.221	6/1208	4/2795	17/3739	5.336	0.069*
**Respondent received COVID-19 therapy** (Yes/No)	157/7612	72/3620	85/3992	0.178	0.673	19/1195	34/2765	104/3652	21.075	0.000*

We collected information about the family structure and occupation of the parents of all participants. A large proportion of participants lived in three-generational households (35.6%) and in families with three individuals including the respondent (33.6%),and lived with single parents (10.2%). Others had different family structures such as living in more than three-generational households (20.7%). The most frequent parental occupations were freelancers (23.7%), self-employed workers (20.4%), migrant workers (20.3%), and farmers (13.8%). A smaller proportion were medical workers (2.1%), police officers (0.7%), civil servants (3.7%), and teachers (2.5%).A total of 165 students had family members who had been diagnosed with COVID-19.Participants were also exposed to the virus via family members who were doctors (272 students) and frontline workers (111 students).

### Total CRIES-13 score

The stress response of participants to the COVID-19 pandemic was measured based on CRIES-13 total score. A total of 1639 (21.1%) students experienced severe symptoms of psychological stress (total score ≥ 30; Table 2). These symptoms were more serious in senior high school students (23.3%) compared to primary students (20.3%) and junior high school students (18.4%) (*χ*^2^ = 23.5, *p* < 0.001). A higher proportion of female students suffered severe psychological stress than male students (22.3% vs 19.7%; *χ*^2^ = 8.03, *p* = 0.005).

**Table 2:** CRIES-13 scores of students, stratified by sex and grade

	Total	Male	Female	***z/x***^***2***^	p	Primary	Junior High	Senior High	***H/x2***	p
	(***n*** = 7769)	(***n*** = 3692)	(***n*** = 4077)			(***n*** = 906)	(***n*** = 2799)	(***n*** = 3756)		
**Median scores**										
Total	20	19	21	−5.739	0.000*	18.00	19.00	21.00	75.512	0.000*
Intrusion factor	8.00	7.00	8.00	−6.759	0.000*	6.00	8.00	8.00	104.141	0.000*
Avoidance factor	4.00	4.00	4.00	−3.146	0.002*	5.00	4.00	4.00	45.492	0.000*
Arousal factor	7.00	6.00	7.00	−8.563	0.000*	5.00	6.00	8.00	183.669	0.000*
**Distribution by total score, n (%)**										
< 30	6130 (78.9)	2964 (80.3)	3166 (77.7)	8.030	0.005*	967 (79.7)	2283 (81.60)	2880 (76.70)	23.503	0.000*
≥ 30	1639 (21.1)	728 (19.7)	911 (22.3)			247 (20.3)	516 (18.40)	876 (23.30)		

To understand the degree of impact of COVID-19, we compared total CRIES-13 scores among primary school, junior high school, and senior high school students using the Kruskal-Wallis H test. We found a significant difference among the three groups (H = 75.512, *p* < 0.001; Table 2); median total CRIES-13 score was the highest for senior high school students (21), followed by the junior high school(19) and primary students (18).

After adjusting the level of significance using Bonferroni correction, a post-hoc comparison found that total CRIES-13 scores were significantly lower for primary school students (Z = -7.469, adjusted *p* < 0.001) and junior high school students(Z = − 6.616, adjusted *p* < 0.001) than for senior high school students. However, total scores were not significantly different between primary school and junior high school students (Z = -2.368, adjusted *p* = 0.054; Table 3). Across all three student groups, female students had higher total scores than males (median 21 vs 19; Z = -5.739, *p* < 0.001; Table 2).

**Table 3:** The post-hoc comparison of CRIES-13 scores by grade

	Primary &Senior High		Junior High&Senior High		Primary&Junior Hig	
	***z***	Adjusted p	***z***	Adjusted p	***z***	Adjusted p
Total	−7.469	0.000*	−6.616	0.000*	−2.368	0.054
Intrusion factor	−10.120	0.000*	−2.463	0.041*	−7.933	0.000*
Avoidance factor	4.327	0.000*	−3.499	0.000*	6.7	0.000*
Arousal factor	−12.382	0.000*	−9.214	0.000*	−5.200	0.000*

### Factor scores on the CRIES-13

We observed significant differences among the three groups of students in intrusion (H = 103.14, *p* < 0.001), arousal (H = 183.669, *p* < 0.001), and avoidance factor scores (H = 45.492, *p* < 0.001) (Table 2). Post-hoc comparisons of the intrusion factor showed that junior high school students had higher scores than primary students (Z = -7.933, adjusted *p* < 0.001), while senior high school students had higher scores than primary students (Z = -10.120, adjusted *p* < 0.001) and junior high school students (Z = -2.463, adjusted *p* = 0.041; Table 3).

Pairwise comparison of the arousal factor showed that junior high school students had higher scores than primary students (Z = -5.200, adjusted *p* < 0.001), and senior high school students had higher scores than primary students (Z = -12.382, adjusted *p* < 0.001) and junior high school students (Z = -9.214, adjusted *p* < 0.001). In contrast, primary school students had higher avoidance factor scores than junior high school students (Z = 6.7, adjusted *p* < 0.001) and senior high school students (Z = 4.327, adjusted *p* < 0.001), and the scores of junior high school students were lower than those of senior high school students (Z = -3.499, adjusted *p* < 0.001;Table 3).

Across all three student groups, we found that female students had higher intrusion factor (Z = -6.76, *p* < 0.001) and arousal factor scores (Z = -3.15, *p* < 0.001) than males, but lower avoidance factor scores (Z = -3.15, *p* = 0.002; Table 2).

### Factors influencing individual stress response

We performed logistic regression to determine the factors affecting total CRIES-13 scores and stress responses. Our results showed that stress response was influenced by the sex of the participant(*p* = 0.024), school grade (*p* = 0.001), past history of psychological counseling (*p* < 0.001), exposure to infection via relatives (*p* = 0.009), and a recent diagnosis of COVID-19 (*p* = 0.006; Table 4). Individuals suffering from cold, fever, cough, nasal congestion, runny nose, sore throat, and diarrhea within 30 days of taking part in the survey also had a heightened stress response (*p* = 0.002).

**Table 4:** Logistic regression analysis to identify factors that influence risk of severe psychological stress in students

Variable	B	SE	Wald chi-square	OR (95% CI)	p
**Sex** (Male/Female)	0.128	0.057	5.113	1.136 (1.017–1.270)	0.024*
**Grade**			13.860		0.001*
**Primary**	−0.146	0.083	3.073	0.864 (0.735–1.017)	0.080
Junior High	−0.234	0.064	13.200	0.940 (0.697–0.898)	0.000*
**Occupation of parents**			16.827		0.051
Medical staff	0.179	0.215	0.689	1.196 (0.784–1.824)	0.406
Police	0.280	0.327	0.732	1.323 (0.697–2.512)	0.392
Civil servant	0.171	0.169	1.029	1.186 (0.853–1.651)	0.310
Teacher	−0.396	0.230	2.957	0.673 (0.429–1.057)	0.085
Freelancer	0.183	0.100	3.341	1.201 (0.987–1.462)	0.068
Farmer	0.328	0.111	8.741	1.388 (1.117–1.725)	0.003*
Researcher	0.754	0.631	1.429	2.126 (0.617–7.325)	0.232
Worker	0.151	0.104	2.135	1.163 (0.950–1.425)	0.144
Self-employed	0.145	0.103	1.958	1.156 (0.944–1.415)	0.162
**Psychological consultations** (Yes/No)	0.646	0.131	24.389	1.908 (1.477–2.466)	0.000*
**Relatives infected by COVID-19** (Yes/No)	0.451	0.173	6.779	1.570 (1.118–2.206)	0.009*
**Respondent had contact with suspected COVID-19 patient** (Yes/No)	−1.514	0.778	3.787	0.220 (0.048–2.206)	0.006*
**Respondent diagnosed with COVID-19** (Yes/No)	1.937	0.703	7.594	1.570 (1.118–1.632)	0.006*
**Respondent received COVID-19 therapy** (Yes/No)	0.119	0.189	0.400	1.127 (0.778–1.632)	0.527
**Respondent had cold, fever, cough etc. in the previous 30 days** (Yes/No)	0.435	0.142	9.344	1.544 (1.169–2.040)	0.002*

## Discussion

In this study, we examined the early effects of the COVID-19 pandemic on the mental and psychological health of 7769 school students in China using the CRIES-13. Based on total CRIES-13 scores, 1639students (21.1%) experienced symptoms of severe psychological stress, indicating an urgent need to understand the impact of such events on the mental health of children and adolescents.

Women are more likely to experience acute stress reactions and to be at higher risk of PTSD than men [25–31]. In addition, women often show higher scores than men on the invasion and avoidance factors of the CRIES-13 [32, 33]. Studies had also found that women showed more active than men in neural networks associated with fear and arousal [34]. This is consistent with our findings.

Some studies have shown that older individuals respond differently to stressful events compared with younger ones [32, 33, 35–39]. Similarly, studies of children exposed to war violence showed older children more vulnerable to stress [40]. Consistent with these results, we found that the largest proportion of students experiencing severe psychological stress were in senior high school. However, a survey of 8236 US children in grades 4–12 at 6 months after the 9/11 attacks found that primary school students (grades 4–5) were at higher risk of post-traumatic stress symptoms than junior and senior high school students [41]. This discrepancy may reflect that different grades of students may have different degrees of stress disorder under the influence of different events. Future research should focus on more different events.

We found that senior high school students had higher scores on arousal and invasion factors on the CRIES-13, but primary school students had higher avoidance factor scores. This suggests that senior high school students are more likely to feel frightened or anxious, experience flashback reactions associated with the event, and manifest symptoms of arousal. The immaturity of the cognitive process in younger children can make them less susceptible to recurring intrusive thoughts and other cognitive impacts of trauma [42, 43]. A maladaptive cognitive style in adolescents and older children may compromise their ability to regulate emotions, rendering them more vulnerable to PTSD [44].

Based on the regression analysis, we found that the occurrence of cold-related symptoms within one month of participating in the survey significantly influenced stress response. Based on studies of the spread of various viruses, psychosocial factors are related to infection rates. C-reactive protein (CRP) is an acute-phase reactant downstream of the pro-inflammatory cytokines released during influenza infection [45]. Studies have shown that a marker of peripheral inflammation, plasma CRP, may be prospectively associated with PTSD symptom emergence, suggesting that inflammation may predispose to PTSD [46]. On the other hand, the increasing number of patients and suspected cases, and the increasing number of outbreak affected provinces and countries have elicited public worry about becoming infected [47]. As we know, the most common symptoms associated with COVID-19 are fever, cough, dyspnea, expectoration, headache, and myalgia or fatigue [48]. This is similar to the symptoms of the common cold [49]. Particularly, the relevance of perceived threat for health and life and the experienced feelings of vulnerability as mediating factors [50]. It was reported that mental health symptoms may have been common during the COVID-19 outbreak among the general population in China, especially among infected individuals, people with suspected infection, and people who might have contact with patients with COVID-19 [51]. This is consistent with our research results.

Although previous studies have explored the impact of the SARS epidemic on mental health, this is the first study addressing the post-traumatic symptoms of COVID-19 on children and adolescents. Using a relatively large sample ranging widely in age, we conducted a cross-sectional study of the psychological stress status of students who were not from Hubei province at one month after the outbreak of COVID-19 in China [19]. However, this may have caused a bias since the participants were selected from schools in certain regions in China, resulting in findings that may not be generalizable across all children and adolescents. In addition, the survey involved substantially more high school students than primary school students. No strict sampling was another bias of our research, but it was really difficult and almost impossible to do so in COVID-19 crisis.

Even though the timing of the survey may help identify participants who require psychological and clinical intervention, the cross-sectional design meant that we could not assess how persistent the post-traumatic stress symptoms are. Besides, the external validity of our survey is limited, the reasons are: Firstly, most of our samples were from Sichuan. Secondly, we did not use strict sampling and used only online self-rating method without structured mental health examination. These may reduce the representativeness and reliability of the results. But it was really difficult to conduct doctor rating scale and structured mental health examination in COVID-19 pandemic crisis. Lastly, our questionnaires were filled in voluntarily. Only those students whose parents would like to let their children fill in would fill in our questionnaire. In this way, parents and students who were not interested in mental health problems couldn’t be included. This, however, is also a feature of this survey as in a natural state, parents and students interested in their mental health were investigated. Our findings also may have some clinical implications for identification of children and adolescents with high risk for psychological stress after COVID-19.

## Conclusions

In conclusion, our results showed that COVID-19 has placed psychological stresses on primary and secondary school students in China. These stresses are more likely to reach severe levels among female students and senior high school students.

## Data Availability

The data that support the findings of this study are available on request from the corresponding author (Li Yin, yli009@163.com). The data are not publicly available due to privacy or ethical restrictions.

## Abbreviations

COVID-19\SARS-CoV-2: Coronavirus Disease 2019
CRIES-13: the Children’s Impact of Event Scale questionnaire
PTSD: Post-Traumatic Stress Disorder
DSM-IV: the Diagnostic and Statistical Manual of Mental Disorders IV
LR: likelihood ratio
CRP: C-reactive protein
